# Effectiveness of a health technician-delivered brief intervention for alcohol: a Bayesian reanalysis of a clinical trial

**DOI:** 10.1186/s13104-022-06071-7

**Published:** 2022-05-16

**Authors:** Nicolas A. Barticevic, Fernando Poblete, Laura Bradshaw, Marcus Bendtsen

**Affiliations:** 1 Department of Family Medicine, School of Medicine, Pontificia Universidad Católica de Chile, Alameda 340, 8331150 Santiago, PO Chile; 2 Department of Public Health, School of Medicine, Pontificia Universidad Católica de Chile, Alameda 340, 8331150 Santiago, PO Chile; 3 Department of Medical and Health Sciences, Linköping University, Linköping, Sweden

**Keywords:** Alcohol brief interventions, Non-professionals, Bayesian analysis

## Abstract

**Objective:**

To reanalyze a clinical trial on the effectiveness of a Brief Intervention (BI) delivered by non-professionals to reduce risky alcohol drinking. Our previous null-hypothesis test of the effects of the BI yielded a ‘non-significant’ p-value, yet remained uninformative. Here we use the Bayesian paradigm which allows for expressing the probability of different effect sizes to better inform public policy decisions.

**Results:**

The posterior probability of the odds of risky drinking at follow-up favored a marked effect of the BI, with 96% of the probability mass being less than OR = 1, and 84% being less than OR = 0.8. Our findings show that there is a high probability that the BI delivered by health technicians lowered risky alcohol use. The posterior distributions of the BI’s effects are presented to help contextualize the evidence for policy making in Chile.

## Introduction

Excessive alcohol drinking is the leading cause of mortality and disability in the Chilean population [1]. To address this issue, a nation-wide preventive program in primary care offers detection and brief interventions (BI) for excessive drinking, provided mainly by Health Technicians (HTs) [2]. These health workers have two and a half years of education and training, and perform routine healthcare tasks such as medical check-ups and measuring patients’ vital signs. As alcohol BIs are delivered by physicians in high income countries [3], less is known about BIs’ effectiveness when provided by non-professional health workers. In this context, we conducted a randomized controlled trial (RCT) to inform policy makers regarding the potential impact of the national program [4]. Our main finding was an OR of 0.6 (CI 0.34–1.05) for risk reduction with a p-value of 0.07, from which we concluded that there was no observed effect attributable to the BI, and thus, the program could make better use of resources by not providing one-to-one HT-led interventions.

This conclusion followed the convention that p-values over 0.05 indicate that a null effect cannot be ruled out; however, this convention is problematic for a number of reasons [5], including that point estimates are very sensitive to single data points, failing to acknowledge that a range of effect sizes cannot be ruled out, and not incorporating previous scientific knowledge and contextual factors for decision-making. Moreover, dichotomizing the evidence as if only two categories existed, efficacious or not efficacious, is not fully informative for policy making, particularly when done based on an arbitrary cutoff. On the contrary, this public health decision should rely on comprehensive criteria that incorporates a nuanced understanding of the possible effects of the brief intervention in order to justify its nation-wide implementation. In fact, the program is being implemented in Chile, so the decisions on maintaining, intensifying, or stopping the program would rather rely on the likelihood that marked effects are being obtained.

The null hypothesis test paradigm (NHTP) offers little help in answering these questions since it typically dichotomizes the evidence instead of presenting the whole picture. These difficulties in analyzing and communicating our results motivated us to redo the analyses from a Bayesian perspective. Inference in the Bayesian framework differs from the classical approach. The parameter being estimated (e.g., OR) is considered a random variable with a probability distribution. Inference starts with a "prior" distribution representing our state of knowledge about that parameter, which is then updated according to the observed data. This updated probability distribution is often called a "posterior" distribution, and it informs about the compatibility of different effect sizes with the observed data [6]. So, the values of the parameter that are more likely according to the data obtain a higher probability, and the converse as well. As a result, the Bayesian framework allows for inductive inference [7] by reporting the probabilities of different effect sizes that may be relevant for decision-making. Here we present the results of a reanalysis of data from this clinical trial using Bayesian inference.

## Main text

### Methods

#### Trial summary

The clinical trial was conducted in five primary care facilities in the city of Santiago—Chile and compared two interventions in people with risky alcohol use (i.e., Alcohol Use Disorders Identification Test (AUDIT) total score between 8 and 15) [8]. One group received an informative pamphlet (n = 168), and the other received the same pamphlet plus a HT-delivered BI based on the contents from the pamphlet (n = 174). The BI was based on motivational interviewing according to the guidelines of the Chilean Ministry of Health [2].

Our outcomes were the AUDIT risk category (primary outcome), and the AUDIT-total and AUDIT-C scores (i.e., sum of items one to three), measured 6 months after enrollment. For practical reasons, the only outcome measurement was a one-year retrospective AUDIT. This minimal outcome collection allowed protocol implementation without changing much the way the program operates in the real world.

#### Previous analysis

All analysis were conducted on participants that completed follow-up (n = 294) using Chi-squared and T-tests accordingly. Then, mixed-effects regressions were used to adjust for demographic variables as fixed-effects (age, sex, and educational level) and the health center as random effects. See the main paper for a detailed description of the trial [4].

#### Current analysis

We used Bayesian inference to estimate the same mixed-effects regression models specified in the original analysis. The alcohol risk status (i.e., primary outcome), was modeled with a Bernoulli distribution with fixed effects for all the covariates, and random intercepts for health centers. The model for the primary outcome is presented in Eq. 1:

1 $$ \begin{gathered} {\text{Risky}}\,{\text{drinking}}\sim {\text{Bernoulli}}\left( {\text{q}} \right) \hfill \\ \log \left( {\frac{q}{1 - q}} \right) = \beta_{1} + \beta_{2} \,{\text{Group}} + \beta_{3} \,{\text{Sex}} + \beta_{4} \,{\text{Education}}\left( {{\text{complete}}} \right) + \beta_{5} \hfill \\ {\text{Education}}\left( {{\text{superior}}} \right) + \beta_{6} \,{\text{Age}} + {\text{C}}\,{\text{Center}} \hfill \\ \beta_{[1 - 6]} \sim {\text{Normal}}\,\left( {0,\,1} \right)\, \hfill \\ {\text{C}}\sim {\text{Normal}}\,\left( {0,\,{\text{sigma\_c}}} \right)\, \hfill \\ {\text{sigma\_c}}\sim {\text{Normal}}\,\left( {0,\,1} \right) \hfill \\ \end{gathered} $$

AUDIT-total and AUDIT-C scores (i.e., secondary outcomes), were modeled with normal distributions with fixed effects for all covariates and random intercepts for health centers. The model for the secondary outcomes is presented in Eq. 2:

2 $$ \begin{gathered} {\text{AUDIT-score}}\sim {\text{Normal}}\left( {{\text{mu}},\,{\text{sigma}}} \right) \hfill \\ {\text{mu}} = \beta_{1} + \beta_{2} \,{\text{Group}} + \beta_{3} \,{\text{Sex}} + \,\beta_{4} \,{\text{Education}}\left( {{\text{complete}}} \right) + \beta_{5} \,{\text{Education}}\left( {{\text{superior}}} \right) + \hfill \\ \beta_{6} \,{\text{Age}} + \beta_{7} \,{\text{AUDIT - score}}\left( {{\text{baseline}}} \right) + {\text{C}}\,{\text{Center}} \hfill \\ \beta_{[1 - 7]} \sim {\text{Normal}}\left( {0,\,1} \right)\, \hfill \\ {\text{sigma}} = {\text{Normal}}\,\left( {0,\,1} \right) \hfill \\ {\text{C}}\sim {\text{Normal}}\,\left( {0,\,{\text{sigma\_c}}} \right)\, \hfill \\ {\text{sigma\_c}}\sim {\text{Normal}}\,\left( {0,\,1} \right) \hfill \\ \end{gathered} $$

Hamiltonian Markov chain Monte Carlo was used for Bayesian inference. For each model, 50,000 iterations were run with 25,000 warm-up iterations in four chains. The analyses were run using R with R-Stan version 2.21.0 on a Mac mini M1.

### Results

**Fig. 1:**
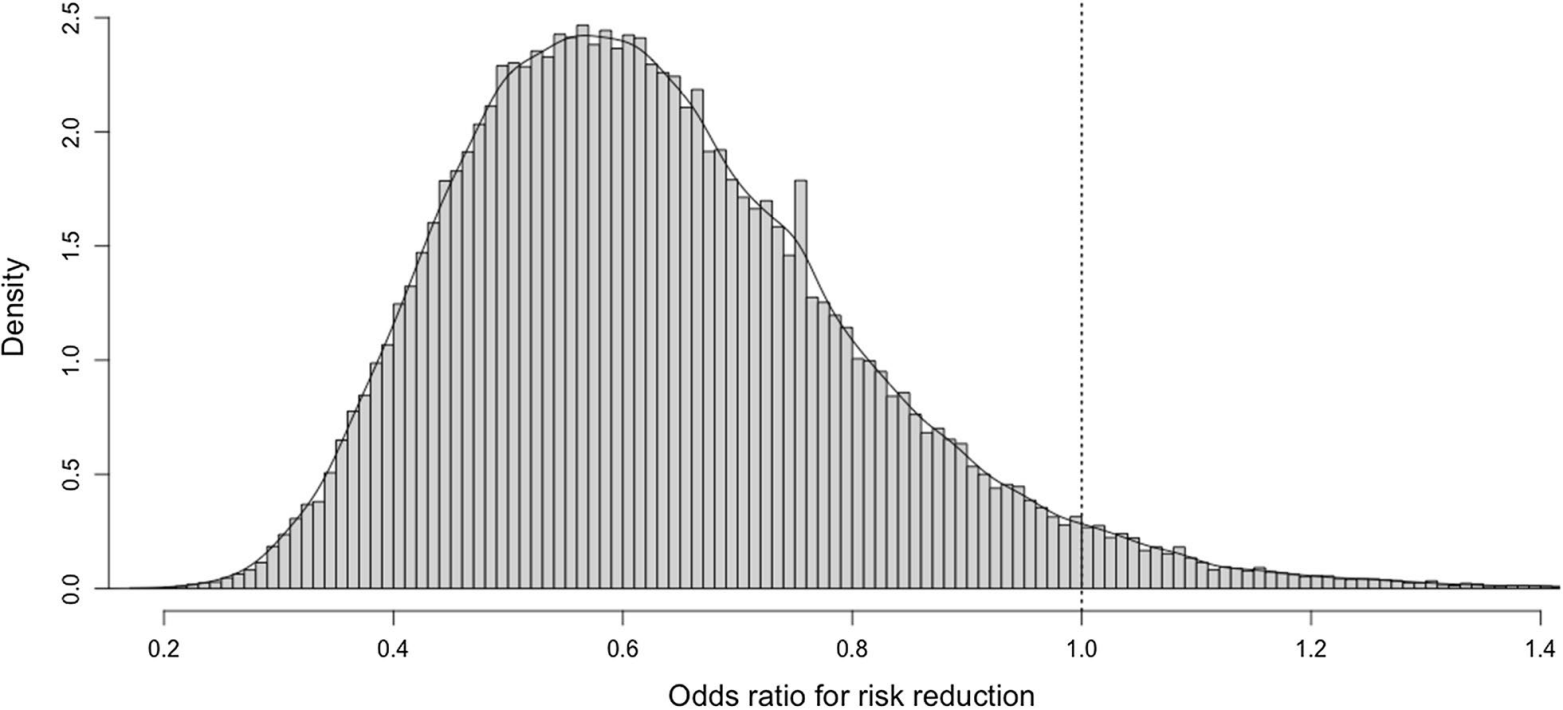
Samples from the posterior distribution of $\beta$_2_ (group): low risk drinking at 6 months follow-up

**Fig. 2:**
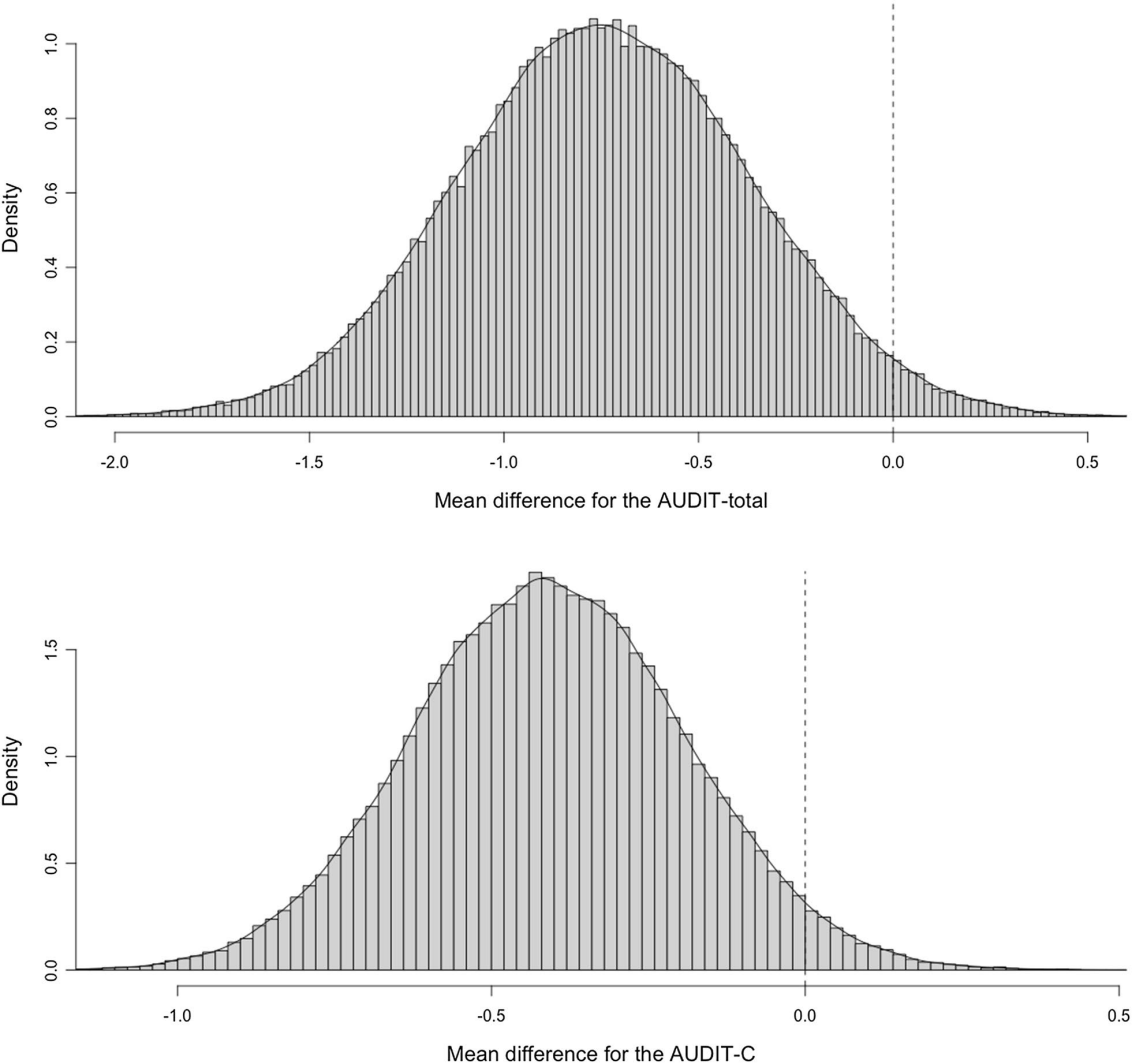
Samples from the posterior distribution of $\beta$_2_ (group): AUDIT scores at 6 months follow-up

The marginal posterior probability distribution for the fixed effect OR of risky drinking is shown in Fig. 1. As can be seen, a strong majority of the distribution is located to the left of the null (i.e., OR = 1), with 96% of the probability mass being less than OR = 1, and 84% being less than OR = 0.8. The mean of the distribution is located at OR = 0.63. Similarly, Fig. 2 shows the marginal posterior probability distributions for the AUDIT scores, where the probability that the difference in means is less that 0 was 98% for the AUDIT-total and 97% for the AUDIT-C. The mean of these distributions is located at MD = 0.74 for the AUDIT-total and at MD = 0.4 for the AUDIT-C. Table 1 shows the posterior probabilities of different effect sizes for both primary and secondary outcomes.

**Table 1:** Cumulative probabilities of possible intervention’s effects

	Posterior probability^a^
Risk reduction^b^	
Odds-ratio < 1	0.96
Odds-ratio < 0.8	0.84
Odds-ratio < 0.6	0.48
AUDIT’s total score^c^	
Any reduction	0.98
At least 0.5 points	0.74
At least 1 point	0.24
AUDIT—C^c^	
Any reduction	0.97
At least 0.25 points	0.77
At least 0.5 point	0.33

^a^Proportion of the posterior probability distribution

^b^Primary outcome. The proportion of participants with low risk (i.e., AUDIT-total < 8), at 6 months follow-up

^c^Secondary outcomes. AUDIT scores at 6 months follow-up

### Discussion

We used Bayesian analysis to reanalyze a clinical trial on the effectiveness of a BI to reduce risky alcohol drinking and found that there was marked evidence suggesting that the intervention reduced risky alcohol use (i.e., most of the posterior probability distribution favored the intervention). In concordance with most evidence on BIs [3, 9], this effect was small but clear; the probability that risky drinking was less in the BI-group compared to the pamphlet group six months post-treatment was 96%. Furthermore, the probability that the OR comparing the two groups was less than 0.8 was 84%. Our findings support the effectiveness of this BI. In the following paragraphs we put these results in the context of our previous analysis and other factors relevant to decision-making for public policy.

First, we address an important difference with respect to scientific inference from the original analysis and this reanalysis. Our current results show the degree to which the data from the trial support the BIs effectiveness, indicating a 96% probability of a positive effect. However, our previous analyses following a NHTP framework obtained a p-value of 0.07; that is, in a hypothetical world where there is exactly no difference between exposure to the BI or the pamphlet, the probability of observing the data from the trial was 7%. Conventionally, this means that a null effect cannot be ruled out, since only extreme observations yielding p-values less than 0.05 are regarded as strong evidence under this paradigm [10]. This usually implies that interventions are ruled ineffective, despite the data supporting a range of effect sizes different from the null. Even though this way of thinking has its merits in protecting us from type 1 error, it also hinders scientific inference when taken as the only criterion to decide if there is an important effect of the intervention. In fact, inference cannot rely only on a statistical model, as recently cautioned by the American Psychology Association [11].

With the Bayesian analysis conducted herein, we obtained a probability distribution over the effects of the BI (in comparison to the pamphlet), allowing us to assess the evidence in a continuous rather than dichotomic manner. This makes it possible to interpret the results in context of existing evidence, specific decision-making contexts, and other available interventions. In our models, we used normal priors centered around the null which encode a conservative view of the effects of the intervention before the data was consulted. Bayesian inference entails updating this prior assumption with the data, migrating to the posterior-probability distributions summarized in Table 1 and shown in Figs. 1 and 2. This means that our effect estimates have been ‘pulled’ towards the null, reducing the risk of spurious findings and reducing the influence of single data points [12]. The posterior distributions show how compatible different effect estimates are with the data collected in the trial, and how unlikely it is that the BI had no effect, or had the effect of increasing risky drinking (probability < 4%).

Another factor for inference is some degree of inaccuracy in our measurements: as this trial was conducted in real world conditions, we refrained from using more precise but demanding instruments. In fact, we used the AUDIT with a one-year retrospective window, which is less sensitive to changes occurred during the last six months (i.e., the study timeframe). However, despite using a one-year retrospective questionnaire, our data strongly indicate that the intervention had an effect.

Altogether, our analyses indicate effectiveness of the BI, and so, the use of HTs as providers is supported by this evidence. Based on this evidence, non-professional health care personal could remain delivering BIs in Chilean primary care: this strategy uses the available health workers to implement a crucial preventive program that otherwise would not be feasible. A related question remains on how these HT-delivered BIs compare in effectiveness to a physician or nurse-delivered BI. Most of the evidence on effectiveness and cost-effectiveness of BIs derives from professional-delivered BIs, so a comparison among both delivery methods would be informative for low- and middle-income countries.

### Conclusion

A health technician-delivered BI reduced risky drinking after six months as compared with the delivery of a pamphlet. The entire posterior distribution of this effect is presented to help contextualize the evidence for policy making in Chile.

## Limitations

A more informative analysis could be perused by incorporating previous knowledge on Brief Interventions (i.e., other prior probability distributions). We used less informative assumptions to remain conservative and comparable to our previous analysis.

## Data Availability

The datasets generated and/or analysed during the current study are available in the Open Science Foundation repository, https://doi.org/10.17605/OSF.IO/AGBH9.

## Abbreviations

AUDIT: Alcohol use disorders identification test
BI: Brief intervention
HT: Health technician
NHTP: Null hypothesis test paradigm
OR: Odds ratio
MD: Mean difference
